# Editorial: Inflammatory bowel disease in children

**DOI:** 10.3389/fped.2026.2016810

**Published:** 2026-10-14

**Authors:** Eylem Sevinc, Yasin Sahin, Nafiye Urganci, Nicole Zeky

**Affiliations:** 1 Department of Pediatric Gastroenterology, Karabuk Universitesi Tip Fakultesi, Karabük, Türkiye; 2 Department of Pediatric Gastroenterology, Gaziantep Islam Science and Technology University, Faculty of Medicine, Gaziantep, Türkiye; 3 Department of Pediatric Gastroenterology, TC Saglik Bakanligi Gaziantep Sehir Hastanesi, Şahinbey, Türkiye; 4 Department of Pediatric Gastroenterology, TC Saglik Bakanligi Sisli Hamidiye Etfal Egitim ve Arastirma Hastanesi, Şişli, Türkiye; 5 Department of Pediatric Gastroenterology, Cincinnati Children’s Hospital Medical Center, Cincinnati, OH, United States

**Keywords:** biologics, children, diagnosis, inflammatory bowel disease, management

The objective of this Research Topic, launched in 2026, was to bring together original research articles, clinical case reports, and comprehensive reviews addressing diverse dimensions of pediatric inflammatory bowel disease (IBD) to expand the current scientific literature. Within the Research Topic “Inflammatory Bowel Disease in Children,” a total of 20 manuscripts were submitted and evaluated, 10 manuscripts were accepted for publication and 10 were rejected following rigorous review by editors and independent reviewers. These contributions address subjects including combined dietary and biologic therapies for accelerating remission, health-related quality of life (HRQoL) assessments across different disease categories, and rare opportunistic infections in immunosuppressed adolescents. Furthermore, the published works explore the clinical evaluation of oral manifestations and alterations in bone structures, gut microbiome profiles associated with responses to exclusive enteral nutrition, and infantile-onset autoinflammatory conditions driven by specific genetic variants. Additional highlights include registry-based analyses on nutritional therapies, atypical extra-intestinal manifestations of IBD, and broader observations regarding therapeutic management approaches.

IBD, encompassing Crohn's disease (CD), ulcerative colitis (UC), and inflammatory bowel disease unclassified (IBD-U), is a chronic, immune-mediated inflammatory disorder of the gastrointestinal tract, thought to arise from an inappropriate mucosal immune response to the intestinal microbiota in genetically susceptible individuals (1). Beyond genetic predisposition and microbial triggers, the pathogenesis of IBD is considered to be multifactorial, involving epithelial barrier dysfunction, dysregulated innate and adaptive immune pathways, and environmental factors that collectively sustain chronic intestinal inflammation (2). In addition, aberrant inflammasome activation has emerged as an important pathogenic mechanism in severe, early-onset autoinflammatory enterocolitis. In particular, germline variants in the NLRC4 gene can drive excessive interleukin-1β-mediated inflammation, contributing to profound intestinal tissue damage (Wang et al.).

The clinical presentation of pediatric IBD spans a wide spectrum, varying by age of onset, disease phenotype, and the location of intestinal involvement. Very early-onset and infant cases frequently manifest with severe, atypical features such as intractable diarrhea, rapid failure to thrive, and extensive perianal disease, whereas older children and adolescents typically present with classic gastrointestinal complaints including abdominal pain, chronic bloody diarrhea, and weight loss alongside extraintestinal manifestations like growth failure and delayed puberty (3). In addition, pediatric CD and UC are frequently associated with prominent extraintestinal manifestations, including peripheral arthritis, primary sclerosing cholangitis, erythema nodosum, and episcleritis. These manifestations may precede the onset of overt gastrointestinal symptoms or occur during disease flares, further complicating the diagnostic process in young patients (4). Moreover, systemic and gastrointestinal signs, pediatric IBD frequently presents with subtle extra-intestinal tissue alterations, as demonstrated by clinical investigations showing that affected children—particularly those with CD—exhibit a higher prevalence of oral mucosal lesions and reduced mandibular trabecular bone complexity evaluated via fractal dimension analysis (Cege et al.). Furthermore, pediatric CD can occasionally manifest through rare extra-intestinal focal infections, such as recurrent vulvar abscesses in adolescents lacking initial gastrointestinal complaints, underscoring the diagnostic challenge of atypical presentations (He et al.).

The diagnostic evaluation of pediatric IBD relies on a careful combination of blood tests, non-invasive stool assays, and advanced imaging modalities. Initial laboratory workup typically includes complete blood counts to check for anemia or leukocytosis, comprehensive metabolic panels, and systemic inflammatory markers such as C-reactive protein and the erythrocyte sedimentation rate (3). Stool-based biomarkers, particularly fecal calprotectin, play an important role in the evaluation of pediatric IBD. As a sensitive and non-invasive marker of neutrophil-driven intestinal inflammation, fecal calprotectin is useful not only in the initial diagnostic assessment but also in monitoring disease activity and treatment response over time. Fecal calprotectin allows clinicians to screen for intestinal inflammation, differentiate IBD from functional gastrointestinal disorders, and closely track mucosal healing or detect subclinical flares during ongoing treatment (5). Furthermore, recent clinical investigations highlight that quantitative dynamic changes in fecal biomarker levels correlate strongly with underlying mucosal healing and long-term therapeutic success, underscoring their clinical utility in guiding personalized management strategies (Zheng and Chen).

For definitive diagnosis and tissue staging, upper gastrointestinal endoscopy and ileocolonoscopy with multiple mucosal biopsies remain the absolute gold standard. Furthermore, to evaluate small bowel involvement and detect deep mural complications without exposing pediatric patients to ionizing radiation, advanced imaging techniques such as magnetic resonance enterography and radiation-free intestinal ultrasound are widely utilized alongside traditional cross-sectional imaging (3, 4).

The management of pediatric IBD has evolved significantly, shifting toward targeted biologic therapies, advanced small molecules, and personalized treatment strategies designed to achieve deep mucosal healing and alter the natural disease course. While conventional therapies such as corticosteroids and immunomodulators remain foundational for induction, biologic agents particularly anti-tumor necrosis factor (anti-TNF) therapies like infliximab and adalimumab are increasingly initiated earlier in high-risk pediatric patients to prevent irreversible bowel damage and growth impairment. Recent therapeutic advancements have expanded the pediatric armamentarium to include newer biologics and advanced targeted agents with distinct mechanisms of action. Vedolizumab, a gut-selective monoclonal antibody, offers an improved safety profile by restricting lymphocyte trafficking specifically to the intestinal mucosa, whereas anti-interleukin-12/23 agents like ustekinumab provide effective alternatives for refractory cases. Furthermore, optimized nutritional therapies such as exclusive enteral nutrition (EEN) remain vital for inducing remission in pediatric CD while minimizing systemic adverse effects in growing children (4). Nonetheless, real-world registry data reveal a substantial implementation gap between guideline recommendations and clinical practice, highlighting that while EEN is heavily endorsed as first-line therapy, adherence to recommended durations often remains limited, and interventions like registry feedback or structured documentation show only modest impacts on closing this gap (Tiachler et al.). Additionally, adjunctive nutritional strategies, such as combining the Crohn's disease exclusion diet (CDED) with partial enteral nutrition alongside first-line biologic therapy, have been associated with more rapid early clinical and biochemical responses. However, real-world data suggest that long-term outcomes, including mucosal healing and fecal calprotectin normalization, may be comparable to those achieved with biologic therapy alone (Audoto et al.). Finally, nationwide healthcare data indicate that pediatric-onset IBD is characterized by a high comorbidity burden and a tendency toward earlier initiation of advanced therapies compared to adult-onset populations, underscoring the critical need to tailor treatment strategies and expand pediatric-approved therapeutic options (Lim et al.).

Pediatric IBD profoundly impairs HRQoL by disrupting the physical, emotional, and social well-being of developing children and adolescents. Growing patients frequently experience psychological distress, chronic fatigue, burdensome treatment routines, and frequent school absenteeism that heavily interfere with daily activities and social development (6). Real-world evaluations assessing disease-specific HRQoL via Inflammatory Bowel Disease Questionnaire (IMPACT)-III, generic well-being via Pediatric Quality of Life Inventory (PedsQL), and family functioning via the PedsQL Family Impact Module demonstrate that while scores remain similar across different disease types or treatment modalities, social functioning scores are highest whereas emotional functioning and parental worry consistently represent the most significant challenges for families (Lım et al.). Consequently, comprehensive pediatric management must extend beyond clinical and biochemical remission to incorporate routine psychosocial screening, validated quality-of-life assessments, and multidisciplinary supportive care.

As we conclude this special issue dedicated to pediatric inflammatory bowel disease, we hope that the diverse studies, clinical insights, and real-world investigations presented herein will prove engaging and valuable to our readers. We believe that these articles, rigorously evaluated through peer review and selected by the editors, contribute to the evolving literature on pediatric IBD by providing broader perspectives on complex clinical management, innovative nutritional and biologic strategies, and the important quality-of-life and family-centered considerations that affect children and their families.
